# Editorial: Comparison of lung cancer and chronic obstructive pulmonary disease in smokers and never-smokers

**DOI:** 10.3389/fimmu.2023.1143288

**Published:** 2023-02-07

**Authors:** Piotr Kuśnierczyk, Elisabeth Taucher, Joanna Domagała-Kulawik

**Affiliations:** ^1^ Laboratory of Immunogenetics and Tissue Immunology, Hirszfeld Institute of Immunology and Experimental Therapy, Polish Academy of Sciences, Wrocław, Poland; ^2^ Department of Internal Medicine, Division of Pulmonology, Medical University Graz, Graz, Austria; ^3^ Maria Curie-Sklodowska Medical Academy, Warsaw, Poland

**Keywords:** editorial, lung cancer, chronic obstructive pulmonary disease, tobacco smoking contribution, smokers, never-smokers

To the memory of Dr. Jerzy Szkudlarek, my best friend, who died of chronic obstructive pulmonary disease. *Piotr Kuśnierczyk*

Tobacco smoking was invented by Native Americans; archaeological evidence dates it to at least 2 300 years ago (1). However, smoking was not an everyday activity in these societies, as tobacco consumption, i.e. pipe smoking, was sacred, and smoking was limited to religious and social ceremonies (1). Many myths are associated with the origin of tobacco and smoking practices (1, 2). Unfortunately, at present, Native Americans are the population with the highest cigarette smoking rate among all ethnic groups in the United States of America, and the only one still growing, albeit slowly and nonsignificantly, in spite of efforts to promote smoking cessation (3).

When America was “discovered” by Europeans, tobacco seeds and the custom of its smoking was imported to the Old Continent. Smoking lost its religious meaning and became an everyday custom leading to addiction, first mostly in men, but within the last 100 years increasingly so in women, particularly in less developed countries. In the years 1950-1952 it was noticed and described that lung cancer is associated with cigarette smoking (4–6). Since then, multiple publications confirming this observation and examining its different aspects have appeared: a PubMed search (Oct 14, 2022, “tobacco smoking and lung cancer”) returns 1,096 results. As the numbers of never-smokers, as well as those having quit smoking increases, particularly in developed countries, the interest in the causes of lung cancer in never-smokers and former smokers is on the rise, and the number of publications on this topic is currently increasing (7), reaching 195 results in a PubMed search for “lung cancer never-smokers”.

A second disease strongly associated with smoking is chronic obstructive pulmonary disease (COPD). This is a progressive respiratory disease manifesting by a largely irreversible airflow limitation due to airway obstruction, being the third leading cause of death (over 3 million) worldwide (8). In addition, both diseases are interconnected to some extent, and COPD has been identified as a risk factor for lung cancer development (9, 10).

Although tobacco smoking is without any doubt the strongest risk factor for both lung cancer and COPD, nevertheless 10-25% of lung cancer cases and 25-30% of COPD cases appear in never-smokers (Taucher et al.). The reasons for this have been widely studied but not completely clarified. In this Research Topic, *Comparison of lung cancer and chronic obstructive pulmonary disease in smokers and never-smokers*, we present three reviews and three original articles discussing differences and similarities between lung cancer and/or COPD in smokers versus never-smokers.

First, Taucher et al. describe the effect of smoking on the immune system, causing chronic inflammation resulting in an increased risk of COPD and lung cancer. They also point out that in never-smokers, other factors such as air pollution, occupational exposure to chemicals, second-hand smoke, pulmonary infections as well as idiopathic pulmonary fibrosis are factors predisposing to COPD, lung cancer or both. A table listing occupational carcinogens for lung cancer gives detailed, valuable information. Of course, all factors contributing to COPD and lung cancer in never-smokers synergistically increase the risk for these diseases in smokers as well.

Further, Wang et al. compare acute exacerbations of COPD (AECOPD) in smokers and never-smokers. They found that recurrent acute exacerbations were more frequent in smokers. Never-smoking patients were, on average, 5 years older, had a higher body mass index, a lower risk of emphysema and lung cancer, and lower levels of circulating eosinophils and basophils. All these findings emphasize the necessity for the cessation of smoking during therapy of patients with AECOPD.

Coming back to lung cancer, de Alencar et al. in their review focused on lung cancer in never-smokers. In contrast to the AECOPD patients mentioned above, lung cancer on average appears earlier in never-smokers than in smokers, but has a better prognosis in never-smokers, partly due to a higher chance of bearing an actionable driver mutation, facilitating targeted therapy. On the other hand, never-smoking patients with lung cancer respond worse to immune-checkpoint inhibitor therapy. The reasons for this may be the low immunogenicity due to a low mutation burden (and resulting paucity of neoantigens which might be presented to CD8+ T cells), high expression of PD-L1 (a target for immune checkpoint inhibitors), immunosuppressive factors, a non-permissive tumor microenvironment, abnormal metabolism, and an impaired organization of the tertiary lymphoid structure. Novel therapeutic approaches are also discussed.

Kuśnierczyk reviewed recent data on the comparison of lung cancer genetics in smokers and never-smokers, including gene expression, mutations, germ-line polymorphisms, sex and ethnic differences, immune response, and therapy. Most of these comparisons speak in favor of treating lung cancer in smokers and never-smokers as two distinct diseases. A characteristic feature differentiating between lung cancer in smokers versus never-smokers are specific somatic mutations: epidermal growth factor receptor (*EGFR*) gene in never-smokers, and K-ras-encoding gene *KRAS*, mutated preferably in smokers. EGFR is a receptor for epidermal growth factor (EGF), and its binding by EGFR initiates several pathways leading to the expression of genes engaged in cell proliferation, prevention of apoptosis etc., ultimately promoting tumor growth. K-ras (Kirsten rat sarcoma virus) is part of the RAS/MAPK pathway. It transmits signals from outside the cell (after binding a ligand, e.g. EGF, by a cell surface receptor, e.g. EGFR) to the cell nucleus. These signals instruct the cell to grow and divide (proliferate) or to mature and take on specialized functions. Mutations of *EGFR* (in never-smokers) or *KRAS* (in smokers) frequently result in constant activation of the cell independently of ligand binding, resulting in unlimited cell proliferation, leading to cancer. Immune mechanisms also differentiate between smokers and never-smokers, as already pointed out by Taucher et al. and de Alencar et al.. All these facts require a different approach to therapy (de Alencar et al., Kuśnierczyk).

The results of Ko et al. are contradicting the principle presented above: these authors were examining uncommon mutations of the *EGFR* gene. They found that these uncommon mutations (i.e., other than exon 19 deletions and L858R mutations), in contrast to common ones, are significantly more frequent in smokers, and complex mutations, combining multiple uncommon and/or common mutations, are even more strongly associated with smoking. Common EGFR mutations usually respond well to targeted tyrosine kinase inhibitors, while therapy response of patients with uncommon mutations is variable. Therefore, the knowledge of which mutations, common or uncommon, prevail in a given tumor, is important for therapy design.

Finally, Andrzejczak et al. present their results on the associations of single nucleotide polymorphisms (SNPs) in the *BTLA* gene, encoding a B and T lymphocyte attenuator, one of immune checkpoints, in non-small cell lung cancer. There were no differences in SNP frequencies or their combinations (haplotypes) between NSCLC-bearing smokers and control individuals. In contrast, there were significant differences between never-smokers with lung cancer and controls, particularly in females who were generally smoking less frequently. This finding suggests that a relatively weak effect of the *BTLA* gene is overdominated in smokers by the potent carcinogenic effects of tobacco smoke, but may contribute to cancer risk in never-smokers significantly. This observation stands in contrast to the effects of polymorphisms of endoplasmic reticulum aminopeptidases on NSCLC risk which were detectable not only in never-smokers but also in smokers, and frequently in opposite directions (11). These enzymes are important in the processing of cancer neoantigens for presentation to CD8+ T cells, and these neoantigens may be different in smokers and never-smokers due to a different mutation burden and profile (Kuśnierczyk, Ko et al.).

We hope that our Research Topic brings a new look at the important question of differences between etiology, pathogenesis and genetics of lung cancer in smokers and never-smokers and, as a result, disparate approaches to therapy of these apparently distinct diseases.

## Author contributions

PK wrote the text. ET an JD-K critically read the text and gave helpful suggestions. All authors contributed to the article and approved the submitted version.
